# Different Concentrations of *Lactobacillus acidophilus* Cell Free Filtrate Have Differing Anti-Biofilm and Immunomodulatory Effects

**DOI:** 10.3389/fcimb.2021.737392

**Published:** 2021-09-13

**Authors:** Rachael M. Wilson, Jean M. Walker, Kingsley Yin

**Affiliations:** Department of Cell Biology and Neuroscience, Rowan University - School of Osteopathic Medicine, Stratford, NJ, United States

**Keywords:** biofilm, probiotics, postbiotics, macrophages, *Pseudomonas aeruginosa*, NF-κB

## Abstract

Probiotics such as various strains of *Lactobacillaceae* have been shown to have antimicrobial and immunomodulatory activity. *In vitro* studies have shown that *Lactobacilli* can decrease bacterial biofilm formation. Effects on immune cells have been unclear with most studies showing anti-inflammatory activity. The mechanism of effects has not been clearly elucidated. In these studies, we used different concentrations of live *Lactobacillus acidophilus* as well as cell free filtrate (CFF) derived from different concentrations of bacteria. Use of CFF is advantageous as a therapeutic because *in vivo* it can directly contact immune cells and its concentration is fixed. Both live cells and CFF inhibited *Pseudomonas aeruginosa* biofilm formation. Importantly, we show that high concentration CFF destroyed mature biofilm. This activity was not due to a lowered pH per se, as pH matched HCl did not remove mature biofilm. High concentration CFF totally inhibited *P. aeruginosa* growth and was bactericidal (>99.99%), but low concentration CFF was not bactericidal. To examine the immunomodulatory effects of *L. acidophilus*, we incubated THP-1 monocytes and derived macrophages with CFF and measured TNFα production. CFF did not significantly increase TNFα production in THP-1 monocytes. When cells were prestimulated with LPS, high concentration CFF increased TNFα production even further. In macrophages, high concentration CFF alone increased TNFα production but did not affect LPS prestimulated cells. In contrast, low concentration CFF decreased TNFα production in LPS prestimulated cells. To elucidate the possible mechanisms for these effects, we repeated the experiments using a NF-κB reporter THP-1 cell line. High concentration CFF increased NF-κB activity in monocytes and macrophages. In LPS prestimulated macrophages, only low concentration CFF reduced NF-κB activity. These results suggest that high concentration CFF alone induced NF-κB expression which could account partially for an increase in TNFα production. On the other hand, in macrophages, the lower non-bactericidal concentration of CFF reduced NF-κB expression and decreased TNFα production after LPS prestimulation. Taken together, the results provide evidence that different concentrations of *L. acidophilus* CFF possess varying bactericidal, anti-biofilm and immunomodulatory effects. This is important *in vivo* to evaluate the possible use of *L. acidophilus* CFF in different conditions.

## Introduction

Bacterial infections are extremely common in the community. The host responds to the infection when bacterial derived products such as lipopolysaccharide (LPS), lipoteichoic acid, peptidoglycans and lipoproteins, all of which possess pathogen associated molecular patterns (PAMPs), bind to pathogen recognition receptors (PRRs) on immune cells. This binding to PRRs on patrolling monocytes and/or tissue macrophages is a critical step in the activation of the innate immune system. The subsequent signaling activates leukocytes to release a large quantity of inflammatory mediators, including chemokines, cytokines, arachidonic acid metabolites, and free radicals. This inflammatory response helps clear bacteria, but if highly elevated, can cause tissue injury or organ failure (Angus and van der Poll, 2013). On the other hand, sustained inflammation may cause dysregulated immune response where the host is unable to clear pathogen (Hotchkiss et al., 2013). Therefore, it is imperative that host defense is stimulated to clear bacteria efficiently without excessive, prolonged activation. Antibiotics are the standard treatment modality for bacterial infections, but overuse of antibiotics can cause antibiotic resistance (Dodds, 2017). Importantly, bacteria have mechanisms such as biofilm formation which allows the bacteria to evade antibiotic action. Biofilm is composed of exopolysaccharides, nucleic acids and proteins that help encapsulate the bacteria and prevents antibiotics and/or host defense from attacking the bacteria (Davies et al., 1998; Costerton et al., 1999; Thornton et al., 2021). Expression of antibiotic resistance genes and/or biofilm formation increases bacterial virulence. In addition, antibiotics have little to no effect on host defense to promote bacteria clearance. Bacteriostatic chloramphenicol or erythromycin have been reported to decrease the innate immune system’s ability to clear bacteria (Kristian et al., 2007). In an ideal situation, an antimicrobial would also be immunomodulatory, to clear bacteria in the most efficient manner.

*P. aeruginosa* is an opportunistic gram-negative bacterium which is of particular concern in immunocompromised patients (Gellatly and Hancock, 2013). The quorum sensing system is a coordinated pathway which increases the virulence of *P. aeruginosa* when the bacteria reach a critical population density. On activation of the quorum sensing system, the bacteria secrete specific autoinducing agents which when bound to their cognate receptors, activate the expression of many virulence genes responsible for the release of exotoxins (pyocyanin and elastase), biofilm formation and antibiotic resistance (Jimenez et al., 2012; Lee and Zhang, 2015).

Probiotics are non-pathogenic bacteria found in the gut microbiome which confer many health benefits on the host. Indeed, probiotics have been reported to have anti-inflammatory properties in rodent models of inflammatory bowel disease (Wang et al., 2020) and Citrobacter- induced colitis (Chen et al., 2009). Supplementation with probiotics has also been reported to have prophylactic benefits against burn wound infection (Argenta et al., 2016), increased survival after administration of multidrug-resistant *P. aeruginosa* (Machairas et al., 2015) and murine sepsis (Khailova et al., 2013). All these studies have administered the probiotic bacteria prophylactically as a pretreatment. These studies, therefore, did not provide information on the possibility of using probiotics as a therapeutic given after the onset of infection. Furthermore, studies are now focusing on postbiotics, which include the substances secreted by probiotic bacteria including proteins and organic acids (Maghsood et al., 2020; Zolkiewicz et al., 2020). In efforts to elucidate cellular mechanisms, studies using probiotics *in vitro* showed interesting results where incubation of macrophages with different *Lactobacilli* strains increased cytokine production (Rocha-Ramirez et al., 2017). It is important to note that for therapeutic purposes probiotics are administered orally. The non-pathogenic bacteria therefore are not in direct contact with immune cells. To confirm that probiotics had immunomodulatory effects, De Marco et al. (2018) incubated *Lactobacilli* cell free supernatants with human macrophages. In these studies using macrophages stimulated with LPS, the *Lactobacilli* appear to be anti-inflammatory where probiotic addition decreased cytokine release (De Marco et al., 2018). Although these studies provide information on the actions of probiotics at the cellular level, they did not attempt to reconcile the differing results. Importantly, none of the aforementioned studies examined the effects of different concentrations of probiotics to investigate the concentration-dependence of the reported effects.

Apart from their effects on immune cells, several studies have reported that various probiotics have direct effects to reduce bacterial virulence. *Lactobacilli* isolated from oral cavities of healthy volunteers reduced biofilm formation and elastase activity (Alexandre et al., 2014). *Lactoplantibacillus plantarum* [*L. plantarum;* (Zheng et al., 2020)] was able to reduce the number of bacteria within human plasma biofilm (Besser et al., 2019). Similarly, a probiotic combination reduced the thickness of a bacterial-fungal polymicrobial biofilm (Hager et al., 2019). *L. plantarum* cell free supernatant reduced *P. aeruginosa* biofilm formation (Ramos et al., 2012). However, there is no work to elucidate if concentrations of probiotic that have cellular modulatory activities also have antimicrobial activity.

The objective of this study was to show that *Lactobacillus acidophilus* (*L. acidophilus*) is both antimicrobial and immunomodulatory. We designed experiments to study the *in vitro* effects of different concentrations of *L. acidophilus* on both monocyte/macrophage activation as well as *P. aeruginosa* growth and biofilm formation/removal. We focused our studies on the effects of cell free filtrates (CFF) derived from different concentrations of *L. acidophilus* bacteria. In addition, we used a NF-κB reporter THP-1 monocyte cell line to elucidate the mechanism by which *L. acidophilus* CFF modulates monocyte/macrophage inflammatory activity.

## Materials and Methods

### Bacterial Strains and Cell Free Filtrate Preparation

All bacterial strains as well as the THP-1 TIB-202 and THP-1 NF-κB-LUC2 cell lines were obtained from the American Type Culture Collection (Manassas, VA, USA)*. L. acidophilus* ATCC 4356™ was cultivated in de Man, Rogosa and Sharpe (MRS) broth (Research Products International, Mt. Prospect, IL, USA) for 48 h at 37°C, 5% CO_2_ as previously described (Khailova et al., 2013; Besser et al., 2019; Han et al., 2019; Maghsood et al., 2020). Prior to use in experiments, cultures were centrifuged at 1920 x g for 6 min. Culture supernatants were discarded and pellets were washed in M63 minimal medium (Amresco, Cleveland, OH, USA) supplemented with 1 mM MgSO_4_, 0.2% glucose, and 0.5% casamino acids (Fisher BioReagents, Pittsburgh, PA, USA) for a total of three washes. After the third wash, pellets were resuspended in M63 minimal medium. To confirm colony forming units (CFUs), cultures were diluted in sterile saline (Molecular Biologicals International, Irvine, CA, USA), spread onto MRS agar plates, incubated for 48 h at 37°C, 5% CO_2_, and colonies counted.

The following preparations of *L. acidophilus* were used: 1) *L. acidophilus* 10^6^ CFU/200 µL, 10^7^ CFU/200 µL or 10^8^ CFU/200 µL (10^6^ – 10^8^ CFU/200 µL) suspended in M63 minimal medium to prepare whole cell suspensions (WCS). 2) To obtain an equivalent CFF, *L. acidophilus* at low, mid, and high concentrations (10^6^ – 10^8^ CFU/200 µL) was cultured for 6 h and then centrifuged at 1920 x g for 6 min. The supernatants were then filtered through a 0.22 µm filter (Pall Corporation, Port Washington, NY, USA). We have data which show that MRS media by itself may inhibit biofilm formation (**Supplemental Data**; **Figure S1**). In our studies, we used a specific quantity of *L. acidophilus* incubated in the same media (M63 minimal medium) as the *P. aeruginosa* for a precise amount of time to obtain cell free filtrate. This methodology affords us the ability to interrogate the pharmacological potency of the CFF.

*P. aeruginosa* ATCC 27853™ was grown on Tryptic soy agar (TSA; Ward’s Scientific, Rochester, NY, USA) overnight at 37°C. Liquid cultures were inoculated by depositing *P. aeruginosa* colonies into Luria-Bertani broth (Gibco: Gaithersburg, MD, USA). The cultures were incubated for 5 h at 37°C with shaking (180 rpm) and then centrifuged for 6 min at 9100 x g. Culture supernatants were removed and pellets were washed three times in M63 minimal medium to promote biofilm formation (Musafer et al., 2014; Mattingly et al., 2018; Wijesinghe et al., 2019; Bullock et al., 2020; Quintieri et al., 2020). The cultures were diluted in M63 minimal medium to OD_600_ between 0.04 - 0.06 using a BioTek Synergy H1 plate reader (Biotek, Winooski, VT, USA). Our studies used M63 minimal medium at pH 5.5 to recapitulate various disease environments that *P. aeruginosa* has been reported to persist in, including on the protective skin barrier (Bullock et al., 2020) and in airways of cystic fibrosis patients (Moriarty et al., 2007).

*Escherichia coli* ATCC BAA-1883™ was grown in Tryptic soy broth (TSB; Sigma-Aldrich, St. Louis, MO, USA) for 24 h at 37°C. The cultures were centrifuged for 6 min at 9100 x g. The culture supernatants were removed and the pellet was washed three times in TSB to remove any culture virulence factors. The culture was then diluted in TSB to OD_600_ 0.06 for planktonic growth studies described below.

### Biofilm Formation Assay

*P. aeruginosa* adjusted to OD_600_ 0.04 was prepared as described above and inoculated into a round-bottom 96-well plate. 100 µL of *L. acidophilus* (10^6^ – 10^8^ CFU/200 µL) WCS or CFF was added together with 100 µL of M63 media making a 50% CFF solution. Each treatment group was performed in quintuplicate. The plate was incubated for 24 h at 37°C. To measure biofilm formation, the plates were washed in phosphate buffered saline (PBS; Growcells, Irvine, CA, USA) three times to remove non-adherent cells. 200 µL of 0.1% crystal violet in ddH20 (Sigma-Aldrich, St. Louis, MO, USA) was added to each well for 15 min. The plates were washed three times in PBS to remove excess crystal violet stain and dried overnight. 200 µL of modified biofilm dissolving solution (10% SDS dissolved in 80% ethanol) (Tram et al., 2013) was added to each well for 15 min. The dissolved biofilm solution was transferred to a flat-bottom 96-well plate and OD_600_ was measured.

### Established Biofilm Assay

To study the effects of *L. acidophilus* WCS or CFF on established biofilm, *P. aeruginosa* biofilms were grown undisturbed for 20 h or 48 h. After incubation, the plates were washed as described above and the biofilms were exposed to M63 media, *L. acidophilus* (10^6^ – 10^8^ CFU/200 µL) WCS or CFF for 1 h or 6 h. We investigated if acidity per se caused the anti-biofilm effects observed. 200 µL of HCl pH-matched to *L. acidophilus* 10^8^ CFU/200 µL and 10^6^ CFU/200 µL CFF (pH 4 and pH 5) were used. In addition, to determine if a protein within the CFF was responsible for biofilm removal, the CFF was boiled (100°C) for 15 min to denature proteins and 200 µL was added to the wells for 6 h. To further investigate protein activity on biofilm removal, 100 µg/mL Proteinase K (Promega, Madison, WI, USA), a broad spectrum serine protease, was reconstituted in 50 mM Tris-HCl (Sigma-Aldrich, St. Louis, MO, USA), 10 mM CaCl_2_ (Fisher Scientific, Fair Lawn, NJ, USA) and added to the CFF for 1 h at 37°C prior to use in experiments. To inhibit protease activity, 1 mM phenylmethylsulfonyl fluoride (PMSF; Bio Basic, Markham, ON, Canada) was added and 200 µL protein digested CFF was added to the well for 6 h. Following treatment incubations, the biofilm biomass was quantified with crystal violet as in biofilm formation assays.

### Planktonic Growth Curves and Bactericidal Activity

*P. aeruginosa* and *E. coli* were prepared as described above. For planktonic growth curve experiments, 100 µL of *P. aeruginosa* or *E. coli* adjusted to OD_600_ 0.06 was added to a flat-bottom 96-well plate. 100 µL of CFF derived from *L. acidophilus* (10^6^ – 10^8^ CFU/200 µL) was added to wells. M63 media was added to make a final volume of 200 µL. This is a 50% CFF solution derived from each quantity of *L. acidophilus* bacteria. To examine if low pH had a significant effect on growth, 100 µL HCl pH-matched to the *L. acidophilus* 10^8^ CFF (pH 4) was used in separate wells. In addition, we utilized 1 µg/mL ciprofloxacin (Enzo, Farmingdale, NY, USA) as positive control. The plate reader was set to 37°C with orbital shaking and programmed to measure OD_600_ every 10 min for 20 h. Following the absorbance measurements, cultures from two wells of each group were recovered and serially diluted in saline. 100 µL of each dilution was spread onto TSA, incubated overnight at 37°C, and colonies counted.

### Inflammatory Mediator Assay

THP-1 (ATCC TIB-202™) monocytes were cultured and maintained in RPMI 1640 with L-glutamine (Corning, Manassas, VA, USA) supplemented with 10% heat-inactivated fetal bovine serum (FBS; Corning, Manassas, VA, USA), 0.05 mM 2-mercaptoethanol (VWR, Solon, OH, USA), and 100 U/mL penicillin G, 100 µg/mL streptomycin (VWR, Solon, OH, USA) at 37°C, 5% CO_2_. To evaluate the effects of *L. acidophilus* CFF on TNFα production, THP-1 monocytes were seeded at 3x10^5^ cells/well in 24-well plates in serum-free and pen-strep free RPMI and incubated overnight. To stimulate TNFα secretion, 50 ng/mL of *E. coli* O111:B4 Lipopolysaccharide (LPS; EMD Millipore Corp., Billerica, MA, USA) was added for 1 h prior to the addition of 50 µL *L. acidophilus* CFF (5% solution) overnight treatment. The cell culture supernatants were collected and stored at -70°C. The levels of TNFα production were also measured in THP-1 monocyte-derived macrophages. Macrophage differentiation was achieved by stimulating THP-1 monocytes with 100 ng/mL phorbol 12-myristate 13-acetate (PMA; Sigma-Aldrich, St. Louis, MO, USA) for 48 h. After differentiation, the media was aspirated and the cells were washed with saline three times. Serum-free and pen/strep- free media was replenished. Following a 24 h rest, the cells were washed with saline and then stimulated with LPS (50 ng/ml) 1 h prior to *L. acidophilus* CFF treatment. THP-1 monocyte and THP-1 monocyte-derived macrophages cell culture supernatants were analyzed for TNFα by ELISA (ELISA; Invitrogen, Carlsbad, CA, USA) according to the manufacturer’s protocols.

### NF-κB Activation Assay

To elucidate the cellular mechanism by which the *L. acidophilus* CFF alters TNFα production, the THP-1 NF-κB-LUC2 (ATCC TIB-202-NFκB-LUC2™) cell line was used. Here, the firefly luciferase gene, *luc2* was placed under the control of the NF-κB promoter such that NF-κB activation could be measured *via* luciferase luminescence. THP-1 NF-κB-LUC2 monocytes and macrophages were cultured in 96-well plates, activated with LPS, and treated with *L. acidophilus* CFF as described in inflammatory mediator assays. In separate experiments, THP-1 NF-κB-LUC2 monocytes were differentiated into macrophages using the methodology noted above. Following overnight treatments, Firefly Luc One-Step Glow Assay Kit (Pierce, Rockford, IL, USA) was used as per the manufacturer’s protocol. Briefly, the cells were lysed and incubated with D-Luciferin substrate for 15 min. The Biotek Synergy H1 plate reader was programmed to measure luminescence with an integration time of 1 sec at 135 gain. In addition, THP-1 monocytes not expressing *luc2* were used as negative controls (**Supplemental Data**; **Figure S2**). To validate the assay, QuantiLum Recombinant Luciferase (Promega, Madison, WI, USA) in Firefly Luc One-Step assay buffer (Pierce, Rockford, IL, USA) with 1% bovine serum albumin (BSA; BioVision, Milpitas, CA, USA) was serially diluted and luminescence measured every 5 min for a 30 min period (**Supplemental Data**; **Figure S2**).

### Statistical Analyses

All data are presented as mean ± s. e. m. and all statistical computations were conducted using GraphPad Prism (San Diego, CA, USA). The area under curves were calculated for planktonic growth curves and statistical significance between groups was tested using one-way ANOVA and Tukey’s multiple comparisons test. For all other studies, data were subjected to one-way ANOVA and Dunnett’s test was used to identify the significance compared to appropriate media controls or LPS stimulated controls. In all statistical analyses, P < 0.05 was regarded as significant.

## Results

### *L. acidophilus* Reduces Biofilm Formation

**Figure 1:**
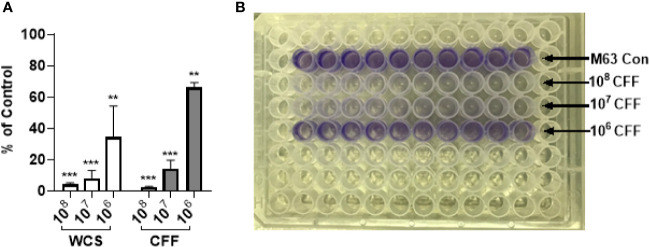
*L. acidophilus* whole cell suspension (WCS) and cell free filtrate (CFF) inhibited *P. aeruginosa* biofilm formation. *P. aeruginosa* cultures were incubated with *L. acidophilus* 10^6^ – 10^8^ CFU/200 µL WCS or CFF at 37°C for 24 h. Apical biofilms were stained with 0.1% crystal violet and absorbance (OD_600_) was measured to quantify biofilm formation. **(A)** All three concentrations of *L. acidophilus* WCS significantly reduced biofilm formation in a concentration-dependent manner. Similarly, all CFF concentrations significantly prevented biofilm formation and demonstrated a dose response. **(B)** Corresponding image of crystal violet stained 96-well plate at the end of biofilm formation experiment. Biofilm data are mean ± s.e.m. percent change from control. **p < 0.01, ***p < 0.001; WCS n = 5 independent experiments; CFF n = 3 independent experiments.

*P. aeruginosa* was incubated in M63 media with *L. acidophilus* WCS (10^6^ – 10^8^ CFU/200 µL), CFF (50% solution as described in Methods) or without any treatment (control) for 24h. Biofilm biomass was then quantitated by crystal violet staining. Both *L. acidophilus* WCS and CFF decreased biofilm formation in a concentration -dependent manner (**Figure 1**). The highest concentrations of *L. acidophilus* WCS and CFF reduced biofilm formation by more than 90% (**Figure 1**). The lowest concentration of CFF derived from 10^6^ CFU/200 µL reduced biofilm formation by approximately 40%, which was similar to WCS. These studies provide evidence that cell to cell contact is not necessary for anti-biofilm activity.

### *L. acidophilus* Removes Established Biofilm

**Figure 2:**
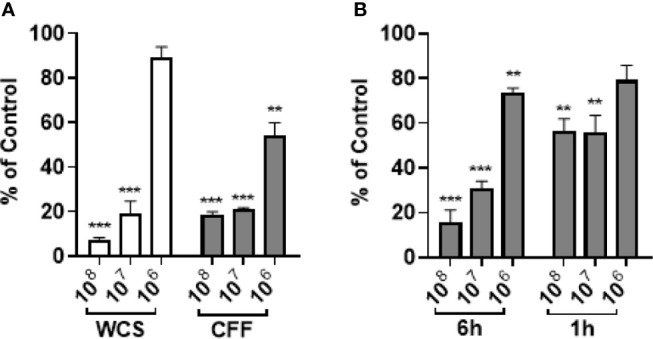
*L. acidophilus* whole cell suspension (WCS) and cell free filtrate (CFF) removed 20 h and 48 h established *P. aeruginosa* biofilm. *P. aeruginosa* was incubated at 37°C for 20 h or 48 h prior to treatment with *L. acidophilus* WCS or CFF for 1 h or 6 h. The remaining biofilm was stained with 0.1% crystal violet and OD_600_ was measured. **(A)** After 6 h treatment, 10^8^ and 10^7^ CFU/200 µL WCS significantly removed 20 h established biofilm, but 10^6^ CFU/200 µL WCS did not. However, all concentrations of CFF significantly abolished 20 h biofilm, though 10^6^ CFF was less effective. **(B)** Following 6 h treatment, all three concentrations of *L. acidophilus* CFF reduced 48 h established *P. aeruginosa* biofilm. Only 10^8^ and 10^7^ CFF treatments significantly removed 48 h biofilm after 1 h treatment. Data are mean ± s.e.m. percent change from control. **p < 0.01, ***p < 0.001; WCS n = 3 independent experiments (20 h biofilm, 6 h treatment); CFF n = 5 independent experiments (20 h biofilm, 6 h treatment); CFF n = 3 independent experiments (48 h biofilm, 1 h and 6 h treatments).

*P. aeruginosa* was allowed to mature for 20 h. After the supernatant was removed and wells washed, WCS (10^6^ – 10^8^ CFU/200 µL), 200 µL CFF or M63 media was added for 6h before the remaining biofilm was quantitated. In these experiments we reasoned that it would be more difficult to remove established biofilm compared to stopping its formation, so we used 100% CFF instead of 50% CFF. WCS at concentrations of 10^7^ and 10^8^ CFU/200 µL significantly removed 20 h established biofilm but 10^6^ CFU/200 µL did not have a significant effect (**Figure 2A**). All concentrations of CFF removed significant amounts of biofilm. In further experiments, we only used CFF as these studies provided evidence that CFF had similar and even better anti-biofilm activity compared to whole cell suspensions. In separate studies, *P. aeruginosa* was allowed to form biofilm for 48 h. Different concentrations of CFF were added to the established biofilm and incubated for 1h and 6 h before the amount of biofilm remaining was measured. After only 1 h, 10^7^ and 10^8^ CFF were able to remove a significant amount of biofilm (**Figure 2B**). After 6 h, 10^7^ and 10^8^ CFF reduced biofilm even further than after only 1 h incubation providing evidence of the time dependence of CFF anti-biofilm activity.

### Anti-Biofilm Effects of *L. acidophilus* Are Not Due to Lower pH Per Se

**Figure 3:**
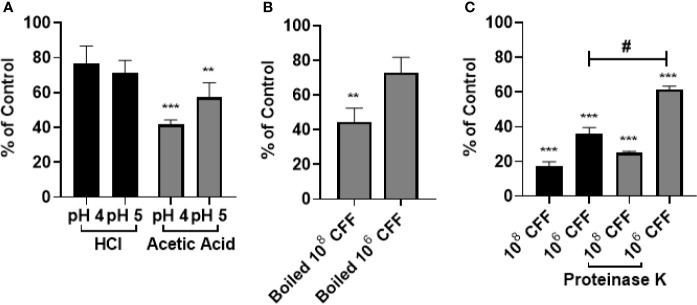
The anti-biofilm effects of *L. acidophilus* cell free filtrate (CFF) were not due to acidity per se. **(A)** HCl and acetic acid pH matched to 10^8^ and 10^6^ CFF (pH 4 and 5, respectively), was added to 20 h established biofilm for 6 h HCl did not reduce *P. aeruginosa* biofilm at either pH tested. Acetic acid (pH 4 and pH 5) removed approximately 60% and 40% of 20 h established biofilm. **(B)** *L. acidophilus* 10^8^ and 10^6^ CFF was boiled at 100°C for 15 min to denature proteins. The boiled CFF was added to 20 h established biofilm for 6 h. 10^8^ boiled CFF significantly reduced established biofilm but 10^6^ CFF did not. **(C)** 100 µg/mL Proteinase K was added to the CFF at 37°C for 1 h and then inhibited with 1 mM PSMF. The protein digested CFF was added to 20 h *P. aeruginosa* biofilm for 6 h. Both concentrations of CFF digested with Proteinase K significantly removed 20 h *P. aeruginosa* biofilm. With low concentration CFF however, Proteinase K reduced the anti-biofilm activity of the CFF. Data are mean ± s.e.m. of percent from control. **p < 0.01, ***p < 0.001 compared to control; ^#^p < 0.05 compared to 10^8^ CFF control; HCl and acetic acid n = 4 independent experiments; boiled CFF n = 3 independent experiments; Proteinase K n = 4 independent experiments.

In these studies, HCl and acetic acid solutions of pH 4 and 5 were incubated with 20 h established biofilms for 6 h. In other wells, established biofilms were also incubated with 100% CFF. **Figure 3A** shows that HCl of pH 4 or 5 did not affect established biofilm. However, acetic acid of pH 4 and pH 5 significantly removed biofilm. In other experiments, the CFF was heated to 100°C for 15 min before incubations. The boiled high concentration CFF was still able to remove a significant amount of established biofilm but the lower concentration CFF was not able to remove a significant amount of pre-formed biofilm (**Figure 3B**). In addition, similar studies utilized Proteinase K, a serine protease, to confirm that proteins within the CFF were not solely responsible for the anti-biofilm effects. **Figure 3C** shows that CFF digested with Proteinase K retains most of its ability to significantly abolish *P. aeruginosa* established biofilm. Taken together, these data indicate that a large protein within the CFF is only partially responsible for the observed anti-biofilm effects.

### *L. acidophilus* CFF inhibits *P. aeruginosa* Growth and Is Bactericidal

**Figure 4:**
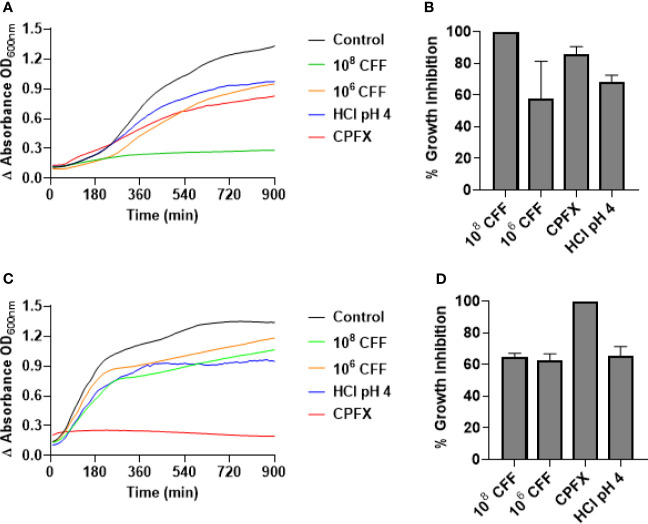
*L. acidophilus* cell free filtrate (CFF) exhibits bactericidal action on *P. aeruginosa* planktonic cell growth but not on *E. coli* growth. Growth curves were conducted by incubating *P. aeruginosa* or *E. coli* in a 96-well plate with *L. acidophilus* 10^8^ or 10^6^ CFF, 1 µg/mL ciprofloxacin (CPFX), or HCl pH 4 at 37°C with orbital shaking. Absorbance (OD_600_) was measured every 10 min for 20 h. **(A)** *L. acidophilus* 10^8^ CFF completely inhibited *P. aeruginosa* planktonic growth (p < 0.001). 10^6^ CFF, HCl pH 4, and 1 µg/mL ciprofloxacin affected *P. aeruginosa* growth, although significantly less effective than 10^8^ CFF. **(B)** At the end of growth curve studies, bacteria were spread on TSA plates and colonies counted. 10^8^ CFF killed > 99.99% *P. aeruginosa* and therefore was bactericidal. However, all other treatments were not bactericidal. **(C)** Both concentrations of *L. acidophilus* CFF and HCl pH 4 did not significantly inhibit *E coli* growth. However, 1 µg/mL ciprofloxacin inhibited *E. coli* growth (p < 0.001). **(D)** Following growth curve studies, *E*. *coli* cells were spread on TSA plates and colonies counted. Only 1 µg/mL ciprofloxacin was bactericidal (> 99.99% inhibition). *P. aeruginosa* growth curves n = 5 independent experiments; *P. aeruginosa* CFUs n = 3 independent experiments; *E. coli* growth curves n = 4 independent experiments; *E. coli* CFUs n = 3 independent experiments.

To test if *L. acidophilus* CFF had direct antimicrobial activity against planktonic bacteria, *P. aeruginosa* or *E. coli* were incubated with CFF (50%) derived from 10^6^ and 10^8^ CFU/200 µL *L. acidophilus* or appropriate controls for 20 h with shaking. At the end of studies, bacteria remaining in the wells were spread on TSA plates. **Figure 4A** shows that high concentration CFF (50% solution) completely inhibited growth of *P. aeruginosa* (**Figure 4A**). To confirm that the high concentration CFF was bactericidal we spread the remaining bacteria on TSA plates. High concentration CFF was completely bactericidal where more than 99.99% of bacteria were killed (**Figure 4B**). Low concentration CFF only partially inhibited growth and was not bactericidal. Under these conditions, 1 µg/ml ciprofloxacin substantially inhibited growth of *P. aeruginosa* but was not bactericidal. In contrast, ciprofloxacin completely inhibited *E. coli* growth and was bactericidal but all concentrations of CFF were not bactericidal towards *E. coli* (**Figures 4C, D**). These results suggest that antimicrobial activity of *L. acidophilus* is species specific.

### *L. acidophilus* CFF Modulates Monocyte/Macrophage Activity

**Figure 5:**
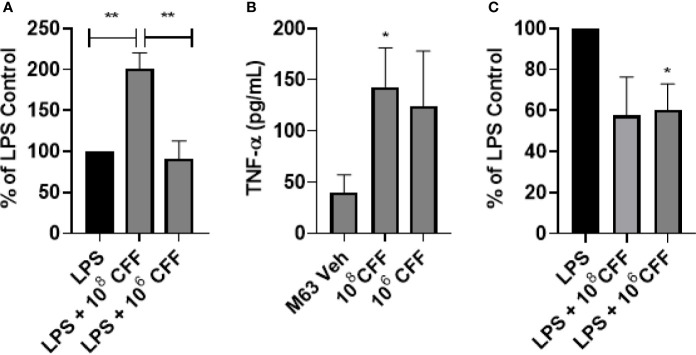
*L. acidophilus* cell free filtrate (CFF) has concentration-dependent effects on TNFα secretion in THP-1 monocytes and monocyte derived macrophages. THP-1 monocytes or monocyte derived macrophages were incubated with *L. acidophilus* 10^8^ or 10^6^ CFF alone or with cells 1 h after LPS stimulation. Supernatants were obtained 20 h after incubation and analyzed for levels of TNFα using ELISA. **(A)** *L. acidophilus* 10^8^ CFF increased TNFα secretion in LPS-stimulated THP-1 monocytes, but 10^6^ CFF had no effect. **(B)** In THP-1 monocyte derived macrophages, *L. acidophilus* 10^8^ CFF alone promoted TNFα production while 10^6^ CFF did not significantly alter TNFα production. **(C)** When THP-1 monocyte derived macrophages were stimulated with 50 ng/mL LPS 1 h prior to *L. acidophilus* CFF treatment, 10^6^ CFF significantly attenuated TNFα production. Data are mean ± s.e.m. % of LPS Control data are mean ± s.e.m. percent change from LPS control adjusted to 100%. *p < 0.05, **p < 0.01 compared to controls; THP-1 monocytes n = 5 independent experiments; THP-1 monocyte derived macrophages n = 5 independent experiments; THP-1 monocyte derived macrophages % of LPS control n = 4 independent experiments.

In these studies, we investigated the immunomodulatory effects of *L. acidophilus* CFF on THP-1 monocytes and THP-1 derived macrophages *in vitro*. As these are mammalian cells which can only grow at a narrow range of pH and temperature, we reasoned that it was imperative to keep the percentage concentration of the CFF solution low. Therefore, for these experiments, CFF percentage solution was 5%. *L. acidophilus* CFF derived from 10^6^ and 10^8^ CFU/200 µL, was incubated with THP-1 monocytes or THP-1 derived macrophages for 20 h. Supernatant was removed for measurement of TNFα. CFF alone did not induce detectable amounts of TNFα (results not shown). In further studies, THP-1 monocytes were stimulated with 50 ng/ml LPS for 1 h before CFF was added. In THP-1 monocytes, LPS caused an increase in TNFα and addition of high concentration CFF increased TNFα further but low concentration CFF had no effect (**Figure 5A**). In THP-1 derived macrophages, high concentration CFF alone significantly increased production of TNFα (**Figure 5B**). When cells were first stimulated with LPS for 1 h, low concentration CFF significantly reduced TNFα production (**Figure 5C**).

### *L. acidophilus* CFF Modulates NF-κB Activity in Monocytes/Macrophages

**Figure 6:**
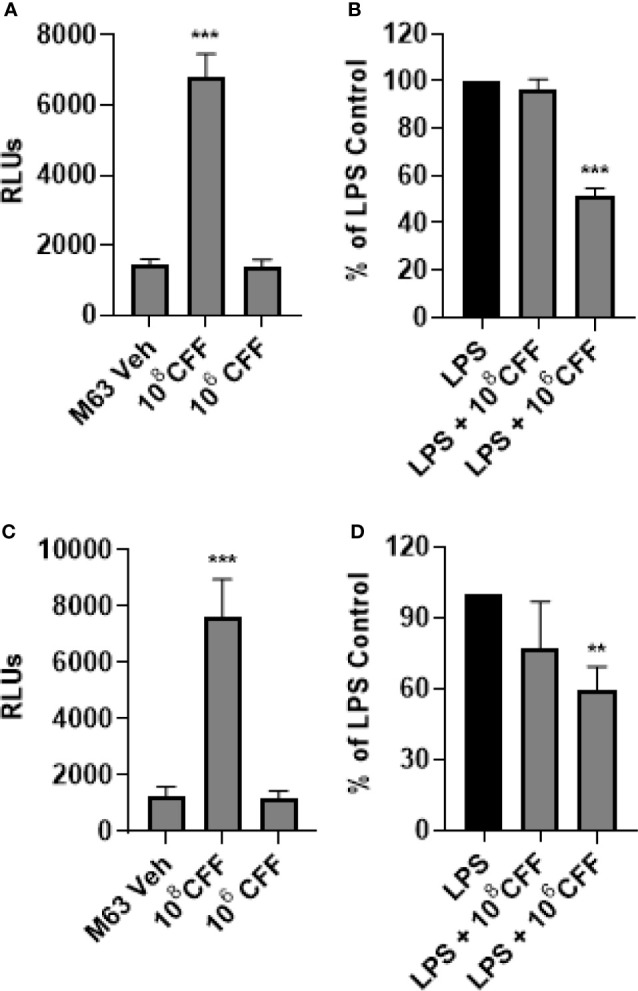
*L. acidophilus* cell free filtrate (CFF) affects NF-κB activity. Using a luciferase NF-κB reporter THP-1 cell line, monocytes and monocyte derived macrophages were incubated with *L. acidophilus* CFF alone or added 1 h after LPS stimulation, for 20 h. Luciferase luminescence was measured to quantify NF-κB activation. **(A)** In monocytes, 10^8^ CFF alone upregulated NF-κB activation but 10^6^ CFF had no effect. **(B)** In LPS stimulated monocytes, only 10^6^ CFF reduced NF-κB activity. **(C)** *L. acidophilus* 10^8^ CFF increased NF-κB activity in monocyte derived macrophages. **(D)** Similar to monocytes, only *L. acidophilus* 10^6^ CFF significantly reduced NF-κB activity in monocyte derived macrophages. Data are relative light units (RLUs) or mean ± s.e.m. percent change from LPS control adjusted to 100%. **p < 0.01, ***p < 0.001 compared to appropriate control; monocytes n = 7 independent experiments; macrophages n = 8 independent experiments.

In these studies, we repeated the previously described experiments using a luciferase NF-κB reporter THP-1 cell line to try to elucidate if NF-κB was involved in *the L. acidophilus* mediated changes in TNFα production. High concentration CFF increased NF-κB activity in THP-1 monocytes as well as macrophages (**Figures 6A, C**). Low concentration CFF alone had no effect on either cell type. After LPS stimulation, high concentration CFF had no significant effect on NF-κB activity in either cell type but low concentration CFF significantly reduced NF-κB activity in both cell types (**Figures 6B, D**).

## Discussion

*L. acidophilus* is a lactic acid producing commensal bacteria found in the gut. It is commonly found in many commercial probiotic drinks and yogurt (Kopp-Hoolihan, 2001; Urbanska et al., 2010; Pei et al., 2017; Pu et al., 2017). Within the gut, *L. acidophilus* poses no serious danger to the host, and in fact has been reported to be important in ensuring that the gut is not overpopulated with potentially pathogenic bacteria (Lievin-Le Moal et al., 2002; Dotan and Rachmilewitz, 2005; Rooks and Garrett, 2016; Plaza-Diaz et al., 2019). As these bacteria live in the luminal border of the gut, it is plausible that their secreted products may regulate both gut bacteria as well as immune cells, including resident macrophages which lie within the gut mucosa and/or monocytes which travel in the blood vessels that supply the gut. In these studies, we show that CFF derived from various concentrations of *L. acidophilus* reduced biofilm formation as well as destroyed established *P. aeruginosa* biofilm. High concentrations of CFF were bactericidal for *P. aeruginosa* but not for *E. coli*. The CFF also modulated inflammatory responses of monocytes and monocyte derived macrophages. CFF alone did not produce detectable TNFα production in monocytes. High concentration CFF alone stimulated macrophages to produce small amounts of TNFα. After LPS stimulation, high concentration CFF stimulated further TNFα production in monocytes. In macrophages, lower concentration CFF reduced LPS-stimulated TNFα production. Using THP-1 cells expressing luciferase under the control of the NF-κB promoter, we show that high concentration CFF increased NF-kB activity in both monocytes and macrophages. Lower concentrations of CFF reduced NF-κB activity in LPS stimulated macrophages, suggesting that the low concentration of CFF reduced production of TNFα by decreasing NF-κB activity.

There are numerous studies in the literature that have shown that various compounds particularly those which inhibit the quorum sensing receptor – LasR (Davies et al., 1998; Savka et al., 2011; Lee and Zhang, 2015) can inhibit virulence. Our studies show however, that quorum sensing inhibition may not be the major mechanism by which *L. acidophilus* reduces *P. aeruginosa* biofilm formation because we show clearly that *L. acidophilus* CFF not only substantially reduced biofilm formation but also dose-dependently killed *P. aeruginosa*. Therefore it is most plausible that the bactericidal action and/or growth inhibition of *L. acidophilus* CFF is responsible for the reduction in formation of *P. aeruginosa* biofilm over time.

With respect to the growth inhibitory action of the CFF, we show that there was a concentration-dependent effect where high concentration CFF inhibited growth and was bactericidal (> 99.99% killing) and low concentration CFF caused approximately 30% growth inhibition. It is important to note that it is not due to acidity per se as pH matched HCl (pH 4) had significantly less growth inhibition than the high concentration CFF. Interestingly, under these conditions, high concentration CFF had a greater inhibitory effect when compared to 1 µg/ml ciprofloxacin. The growth inhibitory effects of *L. acidophilus* CFF were species specific as it did not inhibit pathogenic *E. coli* growth to as great an extent and no concentrations used were bactericidal. These results provide good evidence that *L. acidophilus* secretes products that are growth inhibitory and possess some level of specificity. Such properties may be of use as an efficient antimicrobial with less non-specific adverse effects.

Apart from being able to retard biofilm formation, we show that *L. acidophilus* CFF could also destroy established biofilm. Our results are consistent with the report of (Ramos et al., 2012) which showed that *L. plantarum* supernatant could partially remove 24 h established *P. aeruginosa* biofilm after a further 24 h. Those studies however, did not define the “concentration” of the supernatant nor did they examine other concentrations. Our results show that the capacity for *L. acidophilus* CFF to remove the established biofilm was not due to lowered pH, as pH matched HCl (pH 4 or 5) did not significantly remove the established biofilm. We also performed these experiments after the CFF was heated to 100°C for 15 min and after incubation with Proteinase K. The boiled high concentration CFF retained some of its ability to remove established biofilm but the lower concentration CFF did not have as much anti-biofilm effects as CFF alone, suggesting that a protein may be partially responsible for the effects. Indeed, *Lactobacilli* secrete bacteriocins, which are protein compounds that possess antimicrobial activities against pathogenic bacteria (Kawai et al., 2000). There are multiple reports that demonstrated that bacteriocins secreted from *L. acidophilus* inhibit *Campylobacter jejuni* growth, *Serratia marcescens* and *Bacillus subtilis* planktonic and biofilm growth and *Candida albicans* biofilm (Campana et al., 2012; Vilela et al., 2015; Shahandashti et al., 2016; Sarikhani et al., 2018). The synthesis of bacteriocins is regulated through *L. acidophilus* quorum sensing (Sturme et al., 2007). Specifically, the LuxS/AI-2 quorum sensing system in gram-positive bacteria promotes the transcription of bacteriocin precursors (Kareb and Aïder, 2020). This lends support to our results showing a clear concentration-dependent effect of CFF. Apart from bacteriocins, the active product(s) within the CFF is unclear but it is thought to be organic acids such as acetic acid, butyric acid and lactic acid (Hamad et al., 2017; Al-alousi et al., 2018; Raheem et al., 2018; Rodrigues et al., 2020). The identity of the active compound(s) in the CFF is a subject of active investigation in the laboratory. Taken together, these studies suggest that there does not need to be cell to biofilm contact for *L. acidophilus* to remove established biofilm. The mechanism for the destruction of established biofilm is unclear. It is possible that the CFF time-dependently damages the structure and adhesiveness of the biofilm. It is unlikely that the primary mechanism of CFF to destroy established biofilm is by killing bacteria encased within the biofilm matrix because if this was the primary mechanism we would see first a lack of effect or a relatively small effect at an early time point (1 h) as bacteria are being killed before seeing large destruction of biofilm. Instead we observe as early as 1 h after CFF incubation, 50% of established biofilm is already removed. This argues against the mechanism for destruction of established biofilm being through the bactericidal action of CFF.

As a functional host defense is critical in the fight to clear bacterial infection, we studied the actions of *L. acidophilus* CFF on monocyte and macrophage response. Monocytes are circulating mononuclear cells which have four major functions: phagocytosis, inflammatory cytokine production, antigen presentation and differentiation into macrophages after activation and migration into infected tissue (Jacubzick et al., 2017). Macrophages have functions similar to their precursor monocytes but also participate actively in tissue repair. It is important to note that although the inflammatory response is an important part of host defense to clear pathogens, overzealous response may cause tissue injury. In our studies, the CFF appeared to have different effects on monocytes as compared to macrophages. In monocytes, neither concentration of CFF induced detectable amounts of TNFα but after LPS stimulation, high concentration of CFF increased TNFα production further. In monocyte derived macrophages, high concentration CFF alone increased production of TNFα suggesting that CFF is stimulatory. On the other hand, low concentration CFF decreased TNFα production in LPS stimulated cells which suggests that lower concentration CFF is anti-inflammatory. The results with high concentration CFF alone in macrophages are similar to work by Rocha-Ramirez et al. (2017), while anti-inflammatory results of the lower concentration of CFF are consistent with a report by De Marco et al. (2018). Our results provide evidence that the effects of *L. acidophilus* cell-free filtrate are cell and concentration-dependent where high concentrations of CFF which can remove established biofilm, is bactericidal and is stimulatory of monocytes/macrophages while a lower concentration of CFF which was not bactericidal is anti-inflammatory. The mechanism for this contrasting and unexpected effect has not been elucidated. We speculate that at low concentrations, an anti-inflammatory bioactive compound(s) within the filtrate are present in sufficient quantity to inhibit NF-κB in LPS stimulated cells. On the other hand, at high concentrations of filtrate, certain pro-stimulatory compounds are present in sufficient quantity to overwhelm the effect of anti-stimulatory compounds.

NF-κB is a heterodimeric transcription factor that is implicated in the expression of numerous inflammatory mediators including TNFα (Verstrepen et al., 2008; Vallabhapurapu and Karin, 2009; Wang et al., 2014; Tang and Zhu, 2019; Bhardwaj et al., 2020). Our results using NF-κB reporter THP-1 monocyte-derived macrophages suggest that high concentrations of CFF alone increase TNFα production at least partially by increasing NF-κB activity. In monocytes however, high concentration of CFF increased NF-κB activity without any detectable change in TNFα production. Furthermore, in LPS stimulated monocytes, high concentration CFF increased TNFα release further without further increases in NF-κB activity. Taken together these results suggest that high concentration CFF increases TNFα production only partially by increasing NF-κB activity and other signaling pathways may be involved (Sabio and Davis 2014). In contrast, our results also suggest that the lower concentration of CFF reduced LPS-stimulated TNFα production in macrophages by decreasing NF-κB activity.

In summary, our studies show that different concentrations of *L. acidophilus* secrete products that inhibit growth, reduce biofilm formation, remove established biofilm and modulate monocyte/macrophage responses. High concentrations of *L. acidophilus* CFF were bactericidal, substantially suppressed biofilm formation, effectively removed established biofilm and were stimulatory in monocyte/macrophages. Low concentration CFF was not bactericidal, but reduced biofilm formation, partially removed established biofilm and was anti-inflammatory in macrophages. The studies provide evidence of different properties of specific concentrations of *L. acidophilus* CFF. We believe more research is warranted to ascertain if different concentrations of *L. acidophilus* CFF can be used *in vivo* for different and specific conditions.

## Data Availability Statement

The raw data supporting the conclusions of this article will be made available by the authors, without undue reservation.

## Author Contributions

RW performed all experiments and helped write the manuscript. JW helped design studies and write manuscript. KY helped design the studies and was the principal author of the manuscript. All authors contributed to the article and approved the submitted version.

## Funding

The studies were supported by NIAID (RO1 AI128202) and the New Jersey Health Foundation (PC84-20).

## Conflict of Interest

The authors declare that the research was conducted in the absence of any commercial or financial relationships that could be construed as a potential conflict of interest.

## Publisher’s Note

All claims expressed in this article are solely those of the authors and do not necessarily represent those of their affiliated organizations, or those of the publisher, the editors and the reviewers. Any product that may be evaluated in this article, or claim that may be made by its manufacturer, is not guaranteed or endorsed by the publisher.
