# Supplementary material for: Different Concentrations of Lactobacillus acidophilus Cell Free Filtrate Have Differing Anti-Biofilm and Immunomodulatory Effects
Source: Front Cell Infect Microbiol. 2021 Sep 13;11:737392. doi: 10.3389/fcimb.2021.737392 (PMC8473619; doi:10.3389/fcimb.2021.737392)
Supplement: Supplementary file 1 [file Table_1.docx]

**Supplementary Material**

**Fig. S1.** MRS media inhibits *P. aeruginosa* biofilm formation. MRS media was inoculated with *P. aeruginosa* in a 96-well plate for 24h. 0.1 % crystal violet was used to quantify biofilm formation as previously described for biofilm formation assays. MRS media significantly impacted P*. aeruginosa* biofilm formation and was thus excluded from all other experiments. *p <0.05; n = independent experiments).

**Fig. S2.** Recombinant luciferase luminescence in Pierce Firefly Luc One-Step assay. QuantiLum Recombinant Luciferase was serially diluted in assay buffer with 1 % BSA. Firefly Luc One-Step working solution was added to the wells and luminescence was measured. (A) This data confirms that the Pierce Firefly Luc One-Step Glow assay measures luminescence from firefly luciferase. (B) The diluted recombinant luciferase luminescence was measured over a 20 min period to validate the stability of the signal. This data suggests that the signal remains stable during the span of the experiment. n = 5 independent experiments.
